# {2-Hydr­oxy-6-[(2-oxidophen­yl)imino­methyl-κ^2^ *N*,*O*]phenolato-κ*O* ^1^}phenyl­boron

**DOI:** 10.1107/S1600536810007609

**Published:** 2010-03-06

**Authors:** Araceli Vega, Hugo Tlahuext, Herbert Höpfl

**Affiliations:** a Centro de Investigaciones Químicas, Universidad Autónoma del Estado de Morelos, Av. Universidad 1001, Col. Chamilpa, CP 62209, Cuernavaca Mor., Mexico

## Abstract

The [4.3.0]heterobicyclic title structure, C_19_H_14_BNO_3_, is composed of a five-membered OBNC_2_ ring and a six-membered OBNC_3_ ring, each of which has an approximate envelope conformation. The coordination geometry of the B atom is distorted tetra­hedral. In the crystal structure, centrosymmetrically related mol­ecules are associated through pairs of O—H⋯O hydrogen bonds.

## Related literature

For related boronates, see: Barba *et al.* (2001 ▶); Höpfl *et al.* (1998 ▶); Lamère *et al.* (2006 ▶). For boronates with non-linear optical properties, see: Reyes *et al.* (2005 ▶); Muñoz *et al.* (2008 ▶). For the use of boronates in organic synthesis, see: Rodríguez *et al.* (2005*a* ▶,*b* ▶); López-Ruiz *et al.* (2008 ▶). For a definition of the tetra­hedral character in boron compounds, see: Höpfl *et al.* (1999 ▶).
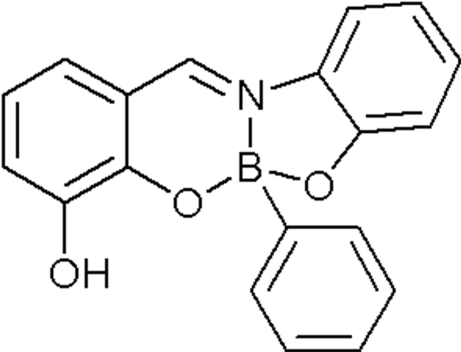


## Experimental

### 

#### Crystal data

C_19_H_14_BNO_3_
*M* *_r_* = 315.12
Orthorhombic, 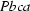

*a* = 14.7245 (13) Å
*b* = 11.196 (1) Å
*c* = 18.4060 (16) Å
*V* = 3034.3 (5) Å^3^
*Z* = 8
Mo *K*α radiation
μ = 0.09 mm^−1^
*T* = 293 K
0.21 × 0.20 × 0.16 mm

#### Data collection

Bruker SMART APEX CCD area-detector diffractometer
Absorption correction: multi-scan (*SADABS*; Sheldrick, 1996 ▶) *T*_min_ = 0.981, *T*_max_ = 0.985
31865 measured reflections
3317 independent reflections
1942 reflections with *I* > 2σ(*I*)
*R*_int_ = 0.088

#### Refinement

*R*[*F* ^2^ > 2σ(*F* ^2^)] = 0.061
*wR*(*F* ^2^) = 0.127
*S* = 1.09
3317 reflections
220 parameters
1 restraint
H atoms treated by a mixture of independent and constrained refinement
Δρ_max_ = 0.14 e Å^−3^
Δρ_min_ = −0.19 e Å^−3^

### 

Data collection: *SMART* (Bruker, 2000 ▶); cell refinement: *SAINT-Plus NT* (Bruker, 2001 ▶); data reduction: *SAINT-Plus NT*; program(s) used to solve structure: *SHELXTL* (Sheldrick, 2008 ▶); program(s) used to refine structure: *SHELXTL*; molecular graphics: *SHELXTL*; software used to prepare material for publication: *PLATON* (Spek, 2009 ▶) and *publCIF* (Westrip, 2010 ▶).

## Supplementary Material

Crystal structure: contains datablocks I, global. DOI: 10.1107/S1600536810007609/tk2636sup1.cif

Structure factors: contains datablocks I. DOI: 10.1107/S1600536810007609/tk2636Isup2.hkl

Additional supplementary materials:  crystallographic information; 3D view; checkCIF report

## Figures and Tables

**Table 1:** Hydrogen-bond geometry (Å, °)

*D*—H⋯*A*	*D*—H	H⋯*A*	*D*⋯*A*	*D*—H⋯*A*
O3—H3′⋯O2^i^	0.84 (2)	1.97 (2)	2.740 (2)	152 (3)
C7—H7⋯O3^ii^	0.93	2.53	3.165 (3)	126

Symmetry codes: (i) 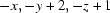; (ii) 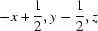.

**Fig. 1.**
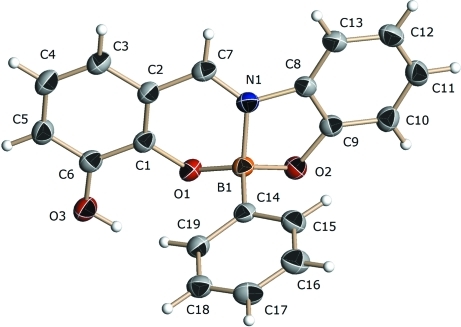
Perspective view of (I), showing the atom-labelling scheme. Displacement ellipsoids are drawn at the 30% probability level and H atoms are shown as small spheres of arbitrary radii.

**Fig. 2.**
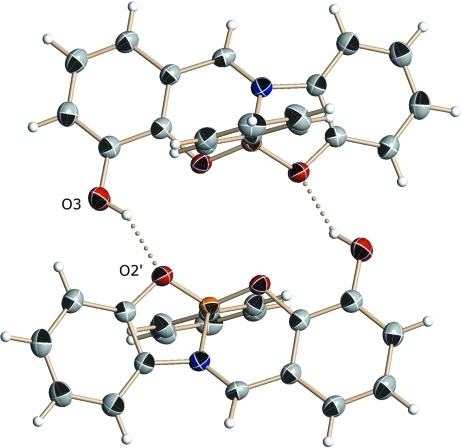
Fragment of the crystal structure of (I), showing the supramolecular aggregate formed through O—H···O hydrogen bonding interactions. Displacement ellipsoids are drawn at the 30% probability level and H atoms are shown as small spheres of arbitrary radii.

## supplementary crystallographic information

### Comment

Recently, boronates derived from ligands having electron-donating and -attracting groups have been prepared in order to generate chromophores with nonlinear optical properties (Reyes *et al.*, 2005; Lamère *et al.*, 2006; Muñoz *et al.*, 2008). Boronates derived from Schiff bases have been applied in Diels-Alder reactions and for the stereoselective addition of Grignard reagents to azomethine carbon atoms (Rodríguez *et al.*, 2005a, 2005b; López-Ruiz *et al.*, 2008).

We have synthesized the [4.3.0]heterobicycle (I), Fig. 1, which is composed of a five-membered OBNC_2_ ring and a six-membered OBNC_3_ ring. The N→B, B—O and B—C bond lengths are comparable to the values determined for the molecular structure of the boronate obtained from the unsubstituted ligand 2-[[(2-hydroxyphenyl)imino]methyl]phenol (Höpfl *et al.*, 1998; Barba *et al.*, 2001); however, the sum of bond lengths at the boron atom is significantly shorter for (I) (6.15 versus 6.18 Å), indicating that there is a small, but systematic tendency for bond length shortening.

The bond angles around the boron atom vary from 98.72 (2) ° for O2—B1—N1 to 112.8 (2) ° for O2—B1—C14, thus indicating a significant distortion from ideal tetrahedral geometry, which can be seen also from the value for the tetrahedral character [71 %] (Höpfl, 1999).

In the five-membered ring, the bond angles vary from 98.7 (2) to 114.0 (2) ° giving a mean value of 106.9 °. In the six-membered heterocycle, the bond angles range from 106.6 (2) to 123.4 (2) °, and the mean value is 117.9 °. Both rings have an approximate envelope conformation, in which the boron atom is localized at the tip. The proximity to an ideal envelope can be illustrated by the N1—C8—C9—O2 torsion angle [-2.7 (3) °] in the first case, and the O1—C1—C2—C7 and C2—C7—N1—B1 torsion angles [2.3 (3) and 2.5 (3) °] in the second case.

The degree of π–delocalization between the aromatic rings connected by the imino group can be analyzed by the C–N–C–C torsion angles (Reyes *et al.*, 2005; Lamère *et al.*, 2006; Muñoz *et al.*, 2008). The C7—N1—C8—C13 torsion angle has a value of 26.1 (4) ° indicating that the π–delocalization is distorted. The distortion can be evidenced also by the relatively long N1—C7 bond [1.282 (3) Å].

In the crystal structure, neighboring molecules are associated through O3—H···O2^i^ hydrogen bonds [symmetry code: (i), -*x*, 2-*y*, 1-*z*] to form supramolecular dimeric units having crystallographic inversion symmetry (Fig. 2). The crystal packing is further stabilized by C—H···O interactions (Table 1).

### Experimental

For the preparation of (I), 2,3-dihydroxysalicylaldehyde (0.250 g, 1.81 mmol), 2-aminophenol (0.198 g, 1.81 mmol) and phenylboronic acid (0.221 g, 1.81 mmol) were dissolved in DMF (35 ml), and the solution was refluxed for 1 h in the presence of a Dean-Stark trap. After cooling, the product precipitated in the form of a yellow crystalline solid, which was filtered and washed with hexane. Yield: 0.102 g (18 %). M.pt. 531 K.

MS (FAB^+^): *m/z* (%) = 316 ([*M+*H]^+^, 40).

### Refinement

H atoms were positioned geometrically and constrained using the riding-model approximation [*C*-H = 0.93 Å, *U*_iso_(H)= 1.2 *U*_eq_(C)]. The hydrogen atom bonded to O3 was located in a difference Fourier map. Its coordinates were refined with a distance restraint: *O*—H = 0.84±0.01 Å and [*U*_iso_(H) = 1.5 *U*_eq_(O)].

### Crystal data

**Table:**

C_19_H_14_BNO_3_	*D*_x_ = 1.380 Mg m^−^^3^
*M**_r_* = 315.12	Melting point: 531 K
Orthorhombic, *P**b**c**a*	Mo *K*α radiation, λ = 0.71073 Å
Hall symbol: -P 2ac 2ab	Cell parameters from 3128 reflections
*a* = 14.7245 (13) Å	θ = 2.2–21.4°
*b* = 11.196 (1) Å	µ = 0.09 mm^−^^1^
*c* = 18.4060 (16) Å	*T* = 293 K
*V* = 3034.3 (5) Å^3^	Rectangular prism, orange
*Z* = 8	0.21 × 0.20 × 0.16 mm
*F*(000) = 1312	

### Data collection

**Table:**

Bruker SMART APEX CCD area-detector diffractometer	3317 independent reflections
Radiation source: fine-focus sealed tube	1942 reflections with *I* > 2σ(*I*)
graphite	*R*_int_ = 0.088
Detector resolution: 8.3 pixels mm^-1^	θ_max_ = 27.0°, θ_min_ = 2.2°
phi and ω scans	*h* = −18→18
Absorption correction: multi-scan (*SADABS*; Sheldrick, 1996)	*k* = −14→14
*T*_min_ = 0.981, *T*_max_ = 0.985	*l* = −23→23
31865 measured reflections	

### Refinement

**Table:**

Refinement on *F*^2^	Primary atom site location: structure-invariant direct methods
Least-squares matrix: full	Secondary atom site location: difference Fourier map
*R*[*F*^2^ > 2σ(*F*^2^)] = 0.061	Hydrogen site location: inferred from neighbouring sites
*wR*(*F*^2^) = 0.127	H atoms treated by a mixture of independent and constrained refinement
*S* = 1.09	*w* = 1/[σ^2^(*F*_o_^2^) + (0.0408*P*)^2^ + 0.7703*P*] where *P* = (*F*_o_^2^ + 2*F*_c_^2^)/3
3317 reflections	(Δ/σ)_max_ = 0.001
220 parameters	Δρ_max_ = 0.14 e Å^−^^3^
1 restraint	Δρ_min_ = −0.19 e Å^−^^3^

### Special details

**Table:**

Geometry. All esds (except the esd in the dihedral angle between two l.s. planes) are estimated using the full covariance matrix. The cell esds are taken into account individually in the estimation of esds in distances, angles and torsion angles; correlations between esds in cell parameters are only used when they are defined by crystal symmetry. An approximate (isotropic) treatment of cell esds is used for estimating esds involving l.s. planes.
Refinement. Refinement of *F*^2^ against ALL reflections. The weighted *R*-factor wR and goodness of fit *S* are based on *F*^2^, conventional *R*-factors *R* are based on *F*, with *F* set to zero for negative *F*^2^. The threshold expression of *F*^2^ > σ(*F*^2^) is used only for calculating *R*-factors(gt) etc. and is not relevant to the choice of reflections for refinement. *R*-factors based on *F*^2^ are statistically about twice as large as those based on *F*, and *R*- factors based on ALL data will be even larger.

### Fractional atomic coordinates and isotropic or equivalent isotropic displacement parameters (Å^2^)

**Table:**

	*x*	*y*	*z*	*U*_iso_*/*U*_eq_	
B1	0.10076 (17)	0.8654 (3)	0.51225 (15)	0.0453 (7)	
N1	0.20017 (12)	0.80938 (17)	0.50322 (10)	0.0460 (5)	
O1	0.11329 (10)	0.98838 (14)	0.53706 (8)	0.0486 (4)	
O2	0.07078 (10)	0.85831 (14)	0.43509 (8)	0.0514 (4)	
O3	0.09544 (12)	1.17706 (17)	0.62833 (11)	0.0672 (6)	
H3'	0.0545 (15)	1.147 (3)	0.6024 (14)	0.101*	
C1	0.17986 (15)	1.0082 (2)	0.58667 (12)	0.0467 (6)	
C2	0.25781 (16)	0.9374 (2)	0.59210 (13)	0.0479 (6)	
C3	0.32673 (17)	0.9695 (2)	0.64063 (14)	0.0579 (7)	
H3	0.3794	0.9238	0.6432	0.069*	
C4	0.31745 (18)	1.0667 (3)	0.68403 (14)	0.0613 (7)	
H4	0.3633	1.0874	0.7164	0.074*	
C5	0.23854 (18)	1.1354 (2)	0.67962 (13)	0.0582 (7)	
H5	0.2319	1.2017	0.7096	0.070*	
C6	0.17066 (16)	1.1066 (2)	0.63186 (13)	0.0518 (6)	
C7	0.26874 (16)	0.8426 (2)	0.54104 (13)	0.0506 (6)	
H7	0.3248	0.8055	0.5353	0.061*	
C8	0.19835 (16)	0.7394 (2)	0.43960 (12)	0.0466 (6)	
C9	0.12224 (16)	0.7743 (2)	0.40089 (13)	0.0482 (6)	
C10	0.10560 (18)	0.7249 (2)	0.33315 (14)	0.0576 (7)	
H10	0.0555	0.7478	0.3057	0.069*	
C11	0.1662 (2)	0.6406 (3)	0.30787 (14)	0.0622 (7)	
H11	0.1565	0.6067	0.2624	0.075*	
C12	0.24025 (19)	0.6050 (3)	0.34753 (14)	0.0619 (7)	
H12	0.2788	0.5465	0.3291	0.074*	
C13	0.25807 (17)	0.6548 (2)	0.41423 (14)	0.0565 (7)	
H13	0.3086	0.6321	0.4412	0.068*	
C14	0.03782 (15)	0.7892 (2)	0.56575 (12)	0.0444 (6)	
C15	0.00739 (17)	0.6756 (2)	0.54791 (15)	0.0599 (7)	
H15	0.0247	0.6426	0.5036	0.072*	
C16	−0.04753 (19)	0.6099 (2)	0.59356 (17)	0.0707 (8)	
H16	−0.0665	0.5338	0.5800	0.085*	
C17	−0.07412 (18)	0.6570 (3)	0.65887 (16)	0.0649 (8)	
H17	−0.1107	0.6128	0.6901	0.078*	
C18	−0.04662 (17)	0.7693 (3)	0.67788 (14)	0.0590 (7)	
H18	−0.0651	0.8021	0.7219	0.071*	
C19	0.00840 (16)	0.8338 (2)	0.63199 (13)	0.0523 (6)	
H19	0.0265	0.9100	0.6459	0.063*	

### Atomic displacement parameters (Å^2^)

**Table:**

	*U*^11^	*U*^22^	*U*^33^	*U*^12^	*U*^13^	*U*^23^
B1	0.0384 (14)	0.0458 (16)	0.0517 (17)	0.0035 (13)	−0.0048 (12)	−0.0004 (14)
N1	0.0408 (11)	0.0516 (12)	0.0455 (12)	−0.0010 (9)	0.0017 (9)	0.0026 (10)
O1	0.0430 (9)	0.0478 (10)	0.0551 (10)	−0.0032 (8)	−0.0085 (8)	0.0019 (8)
O2	0.0503 (10)	0.0558 (11)	0.0482 (10)	0.0042 (8)	−0.0050 (8)	0.0005 (8)
O3	0.0517 (11)	0.0594 (12)	0.0905 (15)	−0.0025 (10)	−0.0096 (10)	−0.0189 (10)
C1	0.0441 (14)	0.0494 (15)	0.0466 (14)	−0.0100 (12)	−0.0008 (12)	0.0056 (12)
C2	0.0442 (14)	0.0526 (15)	0.0469 (14)	−0.0062 (12)	−0.0031 (12)	0.0021 (12)
C3	0.0456 (15)	0.0660 (19)	0.0619 (17)	−0.0046 (13)	−0.0079 (13)	0.0010 (15)
C4	0.0540 (17)	0.0685 (19)	0.0614 (17)	−0.0169 (15)	−0.0099 (13)	0.0012 (15)
C5	0.0594 (17)	0.0547 (17)	0.0605 (17)	−0.0124 (14)	−0.0017 (14)	−0.0047 (14)
C6	0.0471 (15)	0.0507 (16)	0.0577 (16)	−0.0095 (12)	0.0020 (12)	0.0026 (13)
C7	0.0409 (14)	0.0577 (17)	0.0533 (15)	0.0020 (12)	−0.0038 (12)	0.0076 (13)
C8	0.0448 (13)	0.0532 (15)	0.0418 (13)	−0.0043 (12)	0.0058 (11)	0.0010 (12)
C9	0.0461 (14)	0.0488 (15)	0.0496 (15)	−0.0032 (12)	0.0024 (12)	0.0041 (13)
C10	0.0598 (16)	0.0647 (18)	0.0483 (16)	−0.0095 (14)	−0.0046 (13)	0.0020 (14)
C11	0.0699 (19)	0.0683 (19)	0.0484 (15)	−0.0157 (16)	0.0126 (14)	−0.0058 (14)
C12	0.0578 (17)	0.0641 (19)	0.0637 (18)	−0.0029 (14)	0.0166 (15)	−0.0065 (15)
C13	0.0487 (15)	0.0663 (18)	0.0545 (16)	0.0001 (13)	0.0073 (13)	−0.0024 (14)
C14	0.0351 (12)	0.0475 (15)	0.0505 (15)	0.0023 (11)	−0.0046 (11)	0.0014 (12)
C15	0.0570 (16)	0.0528 (17)	0.0700 (18)	−0.0022 (14)	0.0112 (14)	−0.0056 (14)
C16	0.0662 (18)	0.0510 (17)	0.095 (2)	−0.0083 (14)	0.0173 (17)	0.0024 (16)
C17	0.0549 (16)	0.070 (2)	0.0702 (19)	−0.0027 (15)	0.0131 (14)	0.0159 (16)
C18	0.0501 (15)	0.074 (2)	0.0530 (16)	−0.0008 (14)	0.0025 (13)	0.0036 (14)
C19	0.0456 (14)	0.0590 (17)	0.0523 (16)	−0.0047 (12)	−0.0013 (12)	−0.0008 (13)

### Geometric parameters (Å, °)

**Table:**

B1—O1	1.463 (3)	C8—C13	1.375 (3)
B1—O2	1.489 (3)	C8—C9	1.384 (3)
B1—C14	1.599 (4)	C9—C10	1.386 (3)
B1—N1	1.601 (3)	C10—C11	1.380 (4)
N1—C7	1.282 (3)	C10—H10	0.93
N1—C8	1.409 (3)	C11—C12	1.371 (4)
O1—C1	1.358 (3)	C11—H11	0.93
O2—C9	1.362 (3)	C12—C13	1.374 (3)
O3—C6	1.362 (3)	C12—H12	0.93
O3—H3'	0.84 (2)	C13—H13	0.93
C1—C6	1.387 (3)	C14—C19	1.387 (3)
C1—C2	1.399 (3)	C14—C15	1.388 (3)
C2—C3	1.399 (3)	C15—C16	1.379 (3)
C2—C7	1.426 (3)	C15—H15	0.93
C3—C4	1.357 (3)	C16—C17	1.370 (4)
C3—H3	0.93	C16—H16	0.93
C4—C5	1.396 (4)	C17—C18	1.367 (4)
C4—H4	0.93	C17—H17	0.93
C5—C6	1.370 (3)	C18—C19	1.375 (3)
C5—H5	0.93	C18—H18	0.93
C7—H7	0.93	C19—H19	0.93
			
O1—B1—O2	112.7 (2)	C9—C8—N1	106.6 (2)
O1—B1—C14	112.5 (2)	O2—C9—C8	114.0 (2)
O2—B1—C14	112.78 (19)	O2—C9—C10	126.4 (2)
O1—B1—N1	106.61 (18)	C8—C9—C10	119.6 (2)
O2—B1—N1	98.70 (18)	C11—C10—C9	117.5 (3)
C14—B1—N1	112.6 (2)	C11—C10—H10	121.2
C7—N1—C8	128.9 (2)	C9—C10—H10	121.2
C7—N1—B1	123.4 (2)	C12—C11—C10	122.3 (3)
C8—N1—B1	106.65 (18)	C12—C11—H11	118.9
C1—O1—B1	117.06 (18)	C10—C11—H11	118.9
C9—O2—B1	108.21 (18)	C11—C12—C13	120.6 (3)
C6—O3—H3'	112 (2)	C11—C12—H12	119.7
O1—C1—C6	117.6 (2)	C13—C12—H12	119.7
O1—C1—C2	123.2 (2)	C12—C13—C8	117.5 (3)
C6—C1—C2	119.2 (2)	C12—C13—H13	121.3
C1—C2—C3	119.7 (2)	C8—C13—H13	121.3
C1—C2—C7	117.9 (2)	C19—C14—C15	115.9 (2)
C3—C2—C7	122.0 (2)	C19—C14—B1	122.1 (2)
C4—C3—C2	120.6 (3)	C15—C14—B1	122.0 (2)
C4—C3—H3	119.7	C16—C15—C14	122.3 (3)
C2—C3—H3	119.7	C16—C15—H15	118.9
C3—C4—C5	119.4 (2)	C14—C15—H15	118.9
C3—C4—H4	120.3	C17—C16—C15	119.8 (3)
C5—C4—H4	120.3	C17—C16—H16	120.1
C6—C5—C4	121.0 (3)	C15—C16—H16	120.1
C6—C5—H5	119.5	C18—C17—C16	119.6 (3)
C4—C5—H5	119.5	C18—C17—H17	120.2
O3—C6—C5	119.2 (2)	C16—C17—H17	120.2
O3—C6—C1	120.7 (2)	C17—C18—C19	120.0 (3)
C5—C6—C1	120.1 (2)	C17—C18—H18	120.0
N1—C7—C2	119.0 (2)	C19—C18—H18	120.0
N1—C7—H7	120.5	C18—C19—C14	122.4 (2)
C2—C7—H7	120.5	C18—C19—H19	118.8
C13—C8—C9	122.5 (2)	C14—C19—H19	118.8
C13—C8—N1	130.8 (2)		
			
O1—B1—N1—C7	29.0 (3)	C7—N1—C8—C13	26.0 (4)
O2—B1—N1—C7	145.9 (2)	B1—N1—C8—C13	−165.7 (3)
C14—B1—N1—C7	−94.8 (3)	C7—N1—C8—C9	−151.6 (2)
O1—B1—N1—C8	−140.00 (19)	B1—N1—C8—C9	16.7 (2)
O2—B1—N1—C8	−23.1 (2)	B1—O2—C9—C8	−13.6 (3)
C14—B1—N1—C8	96.1 (2)	B1—O2—C9—C10	167.3 (2)
O2—B1—O1—C1	−146.93 (18)	C13—C8—C9—O2	179.4 (2)
C14—B1—O1—C1	84.2 (2)	N1—C8—C9—O2	−2.7 (3)
N1—B1—O1—C1	−39.7 (3)	C13—C8—C9—C10	−1.5 (4)
O1—B1—O2—C9	133.55 (19)	N1—C8—C9—C10	176.3 (2)
C14—B1—O2—C9	−97.7 (2)	O2—C9—C10—C11	180.0 (2)
N1—B1—O2—C9	21.4 (2)	C8—C9—C10—C11	1.0 (4)
B1—O1—C1—C6	−154.4 (2)	C9—C10—C11—C12	0.4 (4)
B1—O1—C1—C2	28.1 (3)	C10—C11—C12—C13	−1.5 (4)
O1—C1—C2—C3	174.7 (2)	C11—C12—C13—C8	1.0 (4)
C6—C1—C2—C3	−2.7 (3)	C9—C8—C13—C12	0.5 (4)
O1—C1—C2—C7	2.3 (3)	N1—C8—C13—C12	−176.8 (2)
C6—C1—C2—C7	−175.1 (2)	O1—B1—C14—C19	−6.9 (3)
C1—C2—C3—C4	2.0 (4)	O2—B1—C14—C19	−135.7 (2)
C7—C2—C3—C4	174.1 (2)	N1—B1—C14—C19	113.6 (2)
C2—C3—C4—C5	−0.4 (4)	O1—B1—C14—C15	171.4 (2)
C3—C4—C5—C6	−0.4 (4)	O2—B1—C14—C15	42.6 (3)
C4—C5—C6—O3	−178.9 (2)	N1—B1—C14—C15	−68.0 (3)
C4—C5—C6—C1	−0.3 (4)	C19—C14—C15—C16	−1.0 (4)
O1—C1—C6—O3	2.8 (3)	B1—C14—C15—C16	−179.4 (2)
C2—C1—C6—O3	−179.6 (2)	C14—C15—C16—C17	0.3 (4)
O1—C1—C6—C5	−175.7 (2)	C15—C16—C17—C18	0.6 (4)
C2—C1—C6—C5	1.8 (3)	C16—C17—C18—C19	−0.9 (4)
C8—N1—C7—C2	164.0 (2)	C17—C18—C19—C14	0.1 (4)
B1—N1—C7—C2	−2.5 (3)	C15—C14—C19—C18	0.8 (3)
C1—C2—C7—N1	−14.9 (3)	B1—C14—C19—C18	179.2 (2)
C3—C2—C7—N1	172.9 (2)		

### Hydrogen-bond geometry (Å, °)

**Table:**

*D*—H···*A*	*D*—H	H···*A*	*D*···*A*	*D*—H···*A*
O3—H3'···O2^i^	0.84 (2)	1.97 (2)	2.740 (2)	152 (3)
C7—H7···O3^ii^	0.93	2.53	3.165 (3)	126

Symmetry codes: (i) −*x*, −*y*+2, −*z*+1; (ii) −*x*+1/2, *y*−1/2, *z*.
